# Mechanisms of resistance to immune checkpoint inhibitors

**DOI:** 10.1038/bjc.2017.434

**Published:** 2018-01-02

**Authors:** Russell W Jenkins, David A Barbie, Keith T Flaherty

**Affiliations:** 1 Division of Medical Oncology, Massachusetts General Hospital Cancer Center, Harvard Medical School, 55 Fruit Street, Boston, MA 02114, USA; 2 Department of Medical Oncology, Dana-Farber Cancer Institute, Boston, MA 02215 USA

**Keywords:** PD-1, PD-L1, CTLA-4, immune checkpoint, innate resistance, acquired resistance, immunotherapy, cancer immunology

## Abstract

Immune checkpoint inhibitors (ICI) targeting CTLA-4 and the PD-1/PD-L1 axis have shown unprecedented clinical activity in several types of cancer and are rapidly transforming the practice of medical oncology. Whereas cytotoxic chemotherapy and small molecule inhibitors (‘targeted therapies’) largely act on cancer cells directly, immune checkpoint inhibitors reinvigorate anti-tumour immune responses by disrupting co-inhibitory T-cell signalling. While resistance routinely develops in patients treated with conventional cancer therapies and targeted therapies, durable responses suggestive of long-lasting immunologic memory are commonly seen in large subsets of patients treated with ICI. However, initial response appears to be a binary event, with most non-responders to single-agent ICI therapy progressing at a rate consistent with the natural history of disease. In addition, late relapses are now emerging with longer follow-up of clinical trial populations, suggesting the emergence of acquired resistance. As robust biomarkers to predict clinical response and/or resistance remain elusive, the mechanisms underlying innate (primary) and acquired (secondary) resistance are largely inferred from pre-clinical studies and correlative clinical data. Improved understanding of molecular and immunologic mechanisms of ICI response (and resistance) may not only identify novel predictive and/or prognostic biomarkers, but also ultimately guide optimal combination/sequencing of ICI therapy in the clinic. Here we review the emerging clinical and pre-clinical data identifying novel mechanisms of innate and acquired resistance to immune checkpoint inhibition.

## Clinical response and resistance to immune checkpoint inhibitors

Monoclonal antibodies targeting co-inhibitory immune checkpoints (e.g., PD-1 and CTLA-4) have demonstrated clinical activity in several malignances, including melanoma, non-small cell lung cancer, renal cell carcinoma, bladder cancer, head and neck squamous cell carcinoma, MSI-high colorectal carcinoma, Merkel cell carcinoma, and Hodgkin lymphoma, and have changed the practice of medical oncology (Pardoll, 2012; Topalian *et al*, 2015; Sharma *et al*, 2017). Immune checkpoint inhibitor therapy has been particularly successful in melanoma, for which approved treatments now include anti-PD-1 (nivolumab and pembrolizumab), anti-CTLA-4 (ipilimumab), and combination anti-PD-1/CTLA-4 regimens (nivolumab–ipilimumab). Long-term survival data for patients with melanoma treated with ipilimumab (anti-CTLA-4) indicates 20% of patients show evidence of continued durable disease control or response 5-10 years after starting therapy (Schadendorf *et al*, 2015). The response rate for melanoma patients treated with pembrolizumab (anti-PD-1) was 33% at 3 years with 70–80% of patients initially responding maintaining clinical response (Ribas *et al*, 2016a). Combination immunotherapy or dual immune checkpoint blockade (anti-PD-1+anti-CTLA-4) has recently shown dramatic response rates in patients with metastatic melanoma (RR 58%); however, half of patients experienced significant toxicity from the treatment regimen (Larkin *et al*, 2015; Postow *et al*, 2015) and survival benefit for this approach has to be demonstrated.

Analysis of clinical trial data can identify three broad populations of patients – (1) those that respond initially and continue to respond (responders), (2) those that fail to ever respond (innate resistance), and (3) those that initially respond but eventually develop disease progression (acquired resistance) (Pitt *et al*, 2016; Restifo *et al*, 2016; O'Donnell *et al*, 2017; Sharma *et al*, 2017). Challenges remain in defining responders and non-responders especially given the heterogeneity in patterns of response that can be seen with ICI. Such heterogeneity can be spatial (i.e., different response in different lesions) and/or temporal (e.g., stable disease followed by progression), manifesting within a given patient as mixed responses, oligometastatic progression, and/or stable disease with isolated progression. Despite these complexities, the ‘tail’ on the survival curve suggests long-term disease control is possible in a significant proportion of patients successfully treated with ICI. This possibility of expanding this long-term clinical benefit to more patients with advanced cancer is thus fueling more focused investigation into the elusive mechanisms of response and resistance to ICI therapy.

## Mechanisms of action of immune checkpoint inhibitors

Mechanisms of innate and acquired resistance to ICI therapy are not fully understood, owing in part to the incomplete understanding of the full complement of clinical, molecular, and immunologic factors associated with clinical response and long-term benefit to ICI therapy. In addition, few immune competent pre-clinical models exist in which tumour regression is induced by ICIs (Zitvogel *et al*, 2016), limiting the ability to recapitulate the diversity of tumour-immune interactions in patients. To frame the discussion of innate (primary) and acquired (secondary) resistance, we must first revisit the model of ‘response’ to ICI to focus on crucial steps that can be inhibited, bypassed, or blocked by the tumour, or co-opted by stromal and immune elements of the tumour microenvironment (TME), to subvert the efforts of the immune system to restrain tumour growth.

Although our understanding of the role of PD-1 and PD-L1 on tumour and immune cells continues to evolve (Juneja *et al*, 2017; Lau *et al*, 2017), it is generally accepted that successful anti-tumour immune responses following PD-1/PD-L1 blockade require reactivation and clonal-proliferation of antigen-experienced T cells present in the TME (O'Donnell *et al*, 2017; Sharma *et al*, 2017). Generation of tumour-reactive CD8 T cells requires successful processing and presentation of tumour-associated peptide antigens by antigen-presenting cells (APCs, e.g., dendritic cells) and recognition of these antigenic peptides displayed by MHC I/II. A unique T-cell receptor recognises MHC-bound tumour antigen providing the first signal for T-cell activation and full T-cell activation follows the engagement of the co-stimulatory CD28 receptor on T cells by B7 on the APC (Schumacher and Schreiber, 2015). Tumour-specific CD8 T cells subsequently differentiate into effector T-cells, undergo clonal expansion, traffic to the TME, and ultimately kill tumour cells displaying tumour-associated antigen on HLA, via release of cytolytic effector molecules (e.g., granzyme A/B and perforin) (O'Donnell *et al*, 2017). For long-term immunologic memory (and presumably durable disease control), a subset of effector T cells must differentiate into effector memory T cells (T_EM_) (Ribas *et al*, 2016b), under the guidance of CD4+ helper T cells and dendritic cells; these are maintained for life and respond to re-challenge with antigen (Harty and Badovinac, 2008; Farber *et al*, 2014).

**Figure 1:**
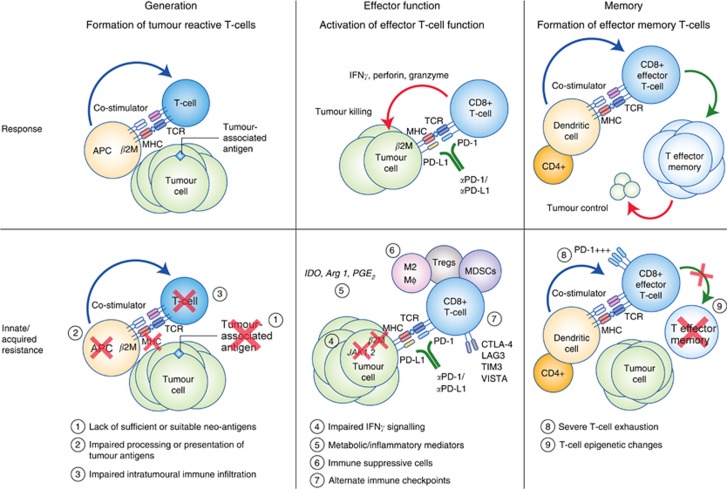
**Response and resistance to ICI therapy.** Upper panel: schematic detailing basic steps involved in generation of tumour-specific T cells, effector T-cell function, and formation of memory T cells. Lower panel: schematic detailing putative mechanisms of innate and/or acquired resistance to ICI therapy.

Failure of ICI therapy can result from defects in any of the steps mentioned above, which can be thought of in three simple categories: (1) insufficient generation of anti-tumour T cells, (2) inadequate function of tumour-specific T cells (Marincola *et al*, 2000; Bronte *et al*, 2005), or (3) impaired formation of T-cell memory (O'Donnell *et al*, 2017; Sharma *et al*, 2017) (Figure 1). Lack of sufficient or suitable neoantigens, impaired neoantigen processing, and/or impaired presentation of neoantigens can all lead to impaired formation of tumour-reactive T cells (O'Donnell *et al*, 2017). Inadequate T-cell function can arise through diverse tumour-intrinsic and tumour-extrinsic immune suppressive components of the TME (Pitt *et al*, 2016), and recent studies have begun to elucidate mechanisms of durable ICI response (Pauken *et al*, 2016; Sen *et al*, 2016). We will explore each of these steps in the context of innate and acquired resistance, as well as strategies to overcome these mechanisms of resistance.

## Innate (primary) and acquired (secondary) resistance

### Insufficient anti-tumour T-cell generation

Tumours can evolve to evade both innate and adaptive arms of the immune system (Gajewski *et al*, 2013), thereby rendering ICI therapy ineffective (Pitt *et al*, 2016; Restifo *et al*, 2016). Tumour-intrinsic mechanisms of immune evasion include genetic and epigenetic alterations to influence neoantigen formation, presentation, and/or processing, as well as alterations in cellular signalling pathways that disrupt the action of cytotoxic T cells (Pitt *et al*, 2016). Tumour-extrinsic mechanisms involve non-cancerous stromal or immune cells, or other systemic influences (e.g., host microbiota) (Joyce and Fearon, 2015; Pitt *et al*, 2016) that can act in concert with cancer cells to promote growth and resistance to ICI.

Successful ICI treatment reactivates T cells directed at tumour-specific mutant proteins (Gubin *et al*, 2014), and lack of suitable neoantigens and alterations in antigen processing and/or presentation is associated with impaired anti-tumour immune response (Schumacher and Schreiber, 2015). Mutational burden is a tumour-intrinsic feature correlated with anti-tumour immune response and response to ICI, presumably by virtue of enhanced neoantigen formation from increased number of non-synonymous single nucleotide variants (Schumacher and Schreiber, 2015; Van Allen *et al*, 2015). Tumour types harbouring high levels of non-synonymous mutations (e.g., melanoma, lung, and bladder) (Lawrence *et al*, 2013) are among those with highest response rates to ICI. Consistent with this notion, DNA-mismatch repair deficiency leading to microsatellite instability is associated with enhanced response to PD-1 blockade (Le *et al*, 2015, 2017).

Importantly, alterations in genes encoding components of the antigen processing and/or presentation apparatus (e.g., class I MHC, β2-microglobulin (B2M)) can also lead to ICI resistance. Downregulation of HLA class I molecules and loss of (B2M) expression have been described. Loss of B2M expression results in impaired cell surface expression of MHC class I, which in turn impairs antigen presentation to cytotoxic T cells (Zaretsky *et al*, 2016). Neoantigen evolution may underlie aspects of acquired resistance (a) via outgrowth of tumour cell clones that never expressed the neo-Ag, despite effective killing of all other clones, or (b) acquisition of genetic changes that result in loss of neo-Ag expression. Although clonal neoantigens are associated with response to ICI therapy (McGranahan *et al*, 2016), evolution of the mutational landscape has been described in patients who developed acquired resistance to ICI (Anagnostou *et al*, 2016).

Strategies to promote immunogenic cell death (e.g., chemotherapy and radiation) or to enhance antigen presentation by stimulating innate immune responses and dendritic cell function (e.g., type I IFN, TLR ligands, LIGHT, and oncolytic viruses) may promote formation or presentation of suitable neoantigens in tumours with a non-inflamed, immune cell poor TME (Pitt *et al*, 2016; O'Donnell *et al*, 2017). In addition, promoting dendritic cell migration, maturation, and function via blockade of immunosuppressive factors (e.g., VEGF, IL-10, and TGF-β), may permit adequate T-cell priming and cooperate with ICI (Pitt *et al*, 2016; O'Donnell *et al*, 2017). HLA-independent tumour killing via natural killer cells (e.g., anti-KIR) (Guillerey *et al*, 2016) may be an option in tumours in which HLA neoantigen presentation is insufficient to promote cytotoxic T-cell killing.

Specific oncogenic signalling pathways can also influence the extent and type of intratumoural immune infiltration. Loss of PTEN is associated with increased levels of CCL2 and VEGF, diminished infiltration of T cells, and resistance to PD-1 blockade (Peng *et al*, 2016), and biallelic PTEN loss was recently reported in an isolated non-responding lesion in a patient with near complete response to PD-1 blockade (George *et al*, 2017). Alterations in *β*-catenin/WNT signalling caused decreased CCL4 production, which led to diminished infiltration of CD103+ dendritic cells and impaired anti-tumour immune responses (Spranger *et al*, 2015). The context of these mutations also influences that type of immune infiltration. For example, loss of STK11/LKB1 in the setting of an oncogenic KRas mutation promotes elaboration of IL-6, which recruits neutrophils, decreases T-cell infiltration, and was associated with higher levels of T-cell exhaustion markers (PD-1, CTLA-4, and TIM3), and lower expression of PD-L1 on tumour cells (Koyama *et al*, 2016a). The recently described innate PD-1 resistance (IPRES) gene signature identified a set of immune suppressive cytokines, EMT transcription factors, and pro-angiogenic factors associated with innate resistance to PD-1 blockade (Hugo *et al*, 2016). Of note, gene signatures enriched in non-responding patients also include signatures for wound-healing, EMT, and treatment/resistance to MAPK pathway inhibition (Hugo *et al*, 2015, 2016). Intriguingly, the receptor tyrosine kinase AXL, whose upregulation is associated with a reversible cell state marked by NF-*κ*B activation and resistance to BRAFi/MEKi (Konieczkowski *et al*, 2014) is a component of the IPRES (Hugo *et al*, 2016). It is tempting to speculate that the IPRES may be part of a multigenic, reversible transcriptional program that could be modulated to influence sensitivity to ICI therapy.

Cell state changes are tumour intrinsic, epigenetic events that often result from reversible chromatin modification through removal or addition of methyl or acetyl marks to DNA or histones. Epigenetic modifying agents (EMAs), including DNA-methyltransferase inhibitors and histone modifiers, can act on tumour cells influencing expression of components of antigen-processing and presentation machinery (e.g., TAP, HLA class molecules, and B2M), novel tumour-associated antigens (e.g., cancer-testis antigens), and cytokines (Heninger *et al*, 2015). Restoration of Th1 cytokine production and enhanced responsiveness to checkpoint blockade has been demonstrated following treatment with DNMT or EZH2 inhibitors (Peng *et al*, 2015). Altered methylation of non-coding regions of the genome may also impact response to immunotherapy. Hypomethylating agents (e.g., 5-aza cytidine) can induce innate immune responses (Roulois *et al*, 2015), influence T-cell priming and effector function, modulate immune suppressive cells within the TME (Kim *et al*, 2014), and enhance response to ICI through induction of endogenous retroviral elements (ERVs) (Chiappinelli *et al*, 2015). Interestingly, tumour-specific ERVs have been associated with anti-tumour cytolytic activity and immune gene cell enrichment (Rooney *et al*, 2015). Although the strength and directionality of this relationship between ERVs and immune infiltration and activation, as well as the role for EMAs as adjuvants for ICI therapy via ERV modulation, requires further investigation, there is growing interest in the role of ERV induction as a mechanism to enhance response to PD-1 blockade (Goel *et al*, 2017).

### Inadequate anti-tumour T-cell effector function

Following successful neoantigen presentation/cross-presentation and T-cell priming, the expanded repertoire of anti-tumour T cells faces an inhospitable TME that may preclude proper T-cell function, thereby limiting the efficacy of ICI therapy (Pitt *et al*, 2016; Sharma *et al*, 2017). These tumour-intrinsic and tumour-extrinsic factors include mutations in key effector pathways, high levels of PD-L1 on tumour cells (and immune cells), high levels of alternate immune checkpoints or co-inhibitory receptors on T cells (e.g., PD-1, CTLA-4), high levels of immune suppressive cytokines or metabolites, and associated recruitment of immune suppressive cells (e.g., myeloid-derived suppressor cells (MDSCs) and regulatory T cells (Tregs)) (O'Donnell *et al*, 2017).

Mutations in immune effector signalling pathways are capable of nullifying the impact of tumour-specific T cells. Whole exome sequencing of tumours from patients that developed resistance following initial clinical response to PD-1 blockade revealed mutations in Janus kinases 1 and 2 (JAK1/JAK2) (Zaretsky *et al*, 2016). These mutations were detected in association with deletion of the wild-type allele resulting in total loss-of-function and loss of interferon responsiveness. This study also described a truncating mutation in B2M, the loss of which resulted in impaired cell surface expression of MHC class I, and defective antigen presentation. The frequency of such mutations appears low based on limited studies to date, but more widespread sequencing may identify additional mutations that lead to innate and/or acquired resistance to ICI therapy (Zaretsky *et al*, 2016; Shin *et al*, 2017). Consistent with these reports of loss-of-function mutations in JAK1/2 as an innate and acquired resistance mechanism, *in vivo* CRISPR screening using a mouse model of melanoma demonstrated that deletion of IFNγ receptors (*Ifrngr1* and *Ifngr2*) and JAK/STAT pathway components (*Jak1*, *Jak2*, and *Stat1*) resulted in resistance to PD-1 blockade (Manguso *et al*, 2017). An intriguing pre-clinical observation in an immune-competent melanoma model is that acquired resistance to ICI blockade could be overcome by inhibiting JAK1/JAK2 signalling, suggesting that JAK/STAT signalling may have a more complex role in mediating response and resistance to ICI (Benci *et al*, 2016). Importantly, the impact of systemic JAK1/2 inhibition with a small molecule inhibitor (e.g., ruxolitinib) almost certainly differs from tumour-specific loss of functional JAK1 and/or JAK2 signalling. Moreover, ICI-resistant cells were derived using anti-CTLA-4 antibody treatment, which promotes Treg depletion (Simpson *et al*, 2013), a property yet to be demonstrated for human anti-CTLA-4 antibodies (e.g., ipilimumab). In light of reports of acquired resistance to cancer immunotherapy through immunoediting (Takeda *et al*, 2017) or acquired resistance via induction of a multigenic resistance programme (Benci *et al*, 2016), further studies will be required to evaluate the impact of interferon signalling as a driver of resistance to ICI therapy.

Within the TME, PD-L1 is constitutively expressed in response to oncogenic signalling, or induced in response to inflammatory cytokines. The physiologic role of immune checkpoints is to maintain self-tolerance and minimise the extent and duration of inflammatory responses, but is co-opted by tumours to promote immune escape via adaptive immune resistance (Keir *et al*, 2008; Pardoll, 2012). Amplification of a region on chromosome 9p24.1 (containing PD-L1, PD-L2, and JAK2) in Hodgkin lymphoma leads to constitutive overexpression of PD-L1 and is thought to explain high clinical response rate to PD-1 blockade (Ansell *et al*, 2015). PD-L1 expression is induced in response to both cell-intrinsic signalling and in response to immune cell-derived soluble factors, such in response to IFN-γ released by effector T cells, and may actually develop in response to T-cell infiltration rather than because of it (Spranger *et al*, 2013). Although intratumoural PD-L1 expression can enrich for responders (e.g., NSCLC) (Reck *et al*, 2016), PD-L1 remains an imperfect biomarker and PD-L1 status neither guarantees nor precludes response to PD-1/PD-L1 blockade (Kluger *et al*, 2017). Although the impact of PD-L1 expression on tumour cells *vs* immune or stromal cells in patients remains unclear, murine studies have confirmed the contribution of PD-L1 on both tumour and immune cells as critical to determine response to PD-1 blockade (Juneja *et al*, 2017; Lau *et al*, 2017). In addition, preliminary evidence in serial tumour biopsies of PD-1 antibody-treated melanoma patients suggests that induction of PD-L1 expression on tumour cells early in the course of therapy improves response prediction (Chen *et al*, 2016).

Functional exhaustion of CD8+ T cells has been well described in chronic viral infections and in cancer, but great heterogeneity exists evidenced by distinct different populations of PD-1^+^ CD8^+^ T cells that respond differently to anti-PD-1 treatment (Blackburn *et al*, 2008; Paley *et al*, 2012). For example, partially exhausted PD-1^+^ CTLA-4^+^ CD8^+^ infiltrating T cells have been correlated with PD-1 response (Daud *et al*, 2016). Exhausted PD-1^+^ CD8^+^ T cells display a distinct chromatin landscape compared with effector T cells and T_EM_ cells (Pauken *et al*, 2016; Sen *et al*, 2016), and these epigenetically distinct T-cell states influence whether or not exhausted PD-1^+^ T cells can be reprogrammed to avoid terminal exhaustion and dysfunction (Philip *et al*, 2017). Evaluation of the specific subsets of CD8^+^ T cells that are expanded in response to PD-1/PD-L1 blockade identified a unique subset of CD8^+^PD-1^+^ T cells that share features of T-follicular helper cells, CD8 memory precursors, and stem cells (Im *et al*, 2016), and resemble CXCR5^+^ CD8^+^ follicular T cells (He *et al*, 2016; Leong *et al*, 2016; Utzschneider *et al*, 2016). Recent profiling of tumour-infiltrating T cells using mass cytometry revealed distinct mechanisms of action of PD-1 and CTLA-4 blockade, demonstrating that PD-1 blockade reinvigorates CD8^+^ T-cell responses, and CTLA-4 blockade results in the expansion of Th1-like CD4^+^ cells expressing the co-stimulatory ligand ICOS (Wei *et al*, 2017). Expression of alternative co-inhibitory immune checkpoints (e.g., CTLA-4, TIM-3, LAG-3, and VISTA) has been associated with resistance to PD-1 blockade (Thommen *et al*, 2015; Koyama *et al*, 2016b), and combination checkpoint blockade using LAG-3^+^PD-1 (Woo *et al*, 2012) and TIM-3^+^PD-1 (Sakuishi *et al*, 2010) has demonstrated improved responses in preclinical models. Although these studies suggest crucial roles for distinct sub-populations of PD-1^+^CD8^+^ T cells, further investigation will be required to determine how to target specific CD8 and CD4 T-cell subsets to overcome primary and acquired resistance.

PD-L1-independent mechanisms of immune escape include alternate immune checkpoints or co-inhibitory receptors, immune suppressive cytokines, immune inhibitory metabolites, and immune suppressive cells (Pitt *et al*, 2016; O'Donnell *et al*, 2017; Sharma *et al*, 2017). Immune suppressive cell types that have been shown to influence ICI efficacy in pre-clinical models include Tregs, MDSCs, Th2 CD4^+^ T cells, and M2-polarised tumour-associated macrophages (Pitt *et al*, 2016; O'Donnell *et al*, 2017; Sharma *et al*, 2017). These cell types individually and collectively promote an immune suppressive TME that prevent anti-tumour cytotoxic and Th1-directed T-cell activities, primarily through the release of cytokines, chemokines, and other soluble mediators (Pitt *et al*, 2016; Sharma *et al*, 2017). Depletion of these immune suppressive cell types (e.g., MDSCs and Tregs) has experimentally been shown to enhance anti-tumour immune responses overcoming innate resistance (Highfill *et al*, 2014; Ngiow *et al*, 2015). Myeloid- and cancer-cell derived indolamine-2,3-dioxygenase (IDO) catabolises tryptophan to the immune suppressive kynurenine (Platten *et al*, 2014). Interestingly, another immune suppressive enzyme, arginase 1, was recently shown to cooperate with the IDO pathway to inhibit dendritic cell function (Mondanelli *et al*, 2017). Recently, tumour-associated macrophages were demonstrated to directly limit PD-1 blockade by removing anti-PD-1 antibodies from PD-1^+^ CD8^+^ T cells in a FcγR-dependent manner (Arlauckas *et al*, 2017). There is also emerging data that additional metabolic (e.g., glucose consumption, lactate production, and cholesterol metabolism) and inflammatory pathways (e.g., cyclooxygenase-2/prostaglandin E2) can simultaneously impact both tumour cells and immune cells (Pitt *et al*, 2016).

### Impaired formation of T-cell memory

The most compelling clinical evidence for the success of ICI relates to the potential for long-term, durable clinical benefit. Thus, although ICI may temporarily re-invigorate CTLs to enhance tumour control, if formation of T_EM_ cells is impaired then clinical response could fade leading to acquired resistance or recurrence of disease following discontinuation of therapy. Expansion of intratumoural T_(EM)_ in response to PD-1 blockade has been demonstrated, and is more pronounced in patients responding to therapy (Ribas *et al*, 2016b), suggesting a key role for T_EM_ cells in anti-PD-1 action and clinical response (O'Donnell *et al*, 2017). The cellular and molecular mechanisms of T_EM_ expansion following PD-1 blockade are not fully understood, however, recent studies have identified distinct transcriptional programmes associated with naive, acute effector, memory, and exhausted T-cell states (Pauken *et al*, 2016; Sen *et al*, 2016). There is emerging evidence that T-cell exhaustion is associated with epigenetic changes that promote a transcriptional landscape distinct from effector or memory CD8 T cells (Sen *et al*, 2016). Importantly, these epigenetic changes appear to limit the durability of CD8 T-cell function following PD-1 blockade (Pauken *et al*, 2016). Reacquisition of memory T-cell response may be limited during conditions if tumour antigen persists, as occurs in patients with higher tumour burden (Huang *et al*, 2017), indicating that future efforts to augment existing new T-cell responses or prime new populations of T cells may be required to generate durable anti-tumour T-cell memory.

## Summary and future directions

**Figure 2:**
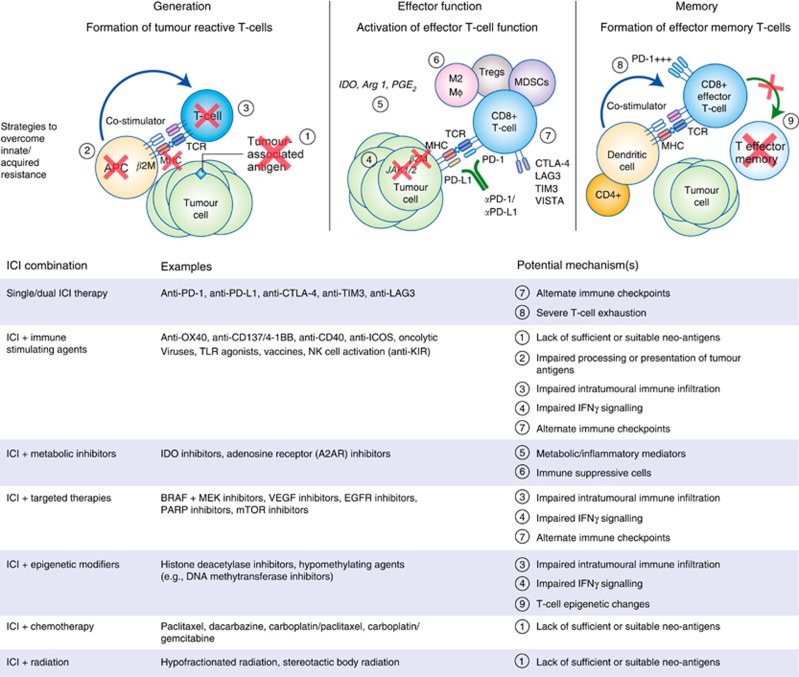
**Combination therapies to overcome resistance to ICI therapy.** Putative mechanisms of innate and/or acquired resistance to ICI therapy. Table listing select approaches for ICI combination therapeutic approaches (Sharma *et al*, 2017).

Several clinical trials of combinations of immunotherapeutic agents with targeted agents, cytotoxic chemotherapy, and/or radiation are underway, all in the effort to provide long-lasting disease control to more patients (Buque *et al*, 2015) (Figure 2). Combination therapies to overcome innate resistance by targeting putative mechanisms of immune evasion within the TME are in various stages of development (Spranger and Gajewski, 2013; Smyth *et al*, 2016). Cancer vaccines (Ott *et al*, 2017; Sahin *et al*, 2017) are showing promise as a means of personalising cancer immunotherapy and potentially enhancing immune memory. Additional research efforts are underway to identify biomarkers associated with resistance and response to ICI, in parallel with early phase clinical testing of novel immune modulatory agents and novel combinations of immune modulators and ICI with other cancer therapies (Mahoney *et al*, 2015).

To date, precision medicine has largely been synonymous with use of molecular and largely genomic features of the tumour to administer specific targeted small molecules and biologics; however, novel and functional precision medicine platforms may offer additional opportunities to tailor therapies for individual patients or patient populations (Friedman *et al*, 2015). As the mechanisms of response and resistance to immune checkpoint inhibitors are further elucidated, molecular and functional technologies can and should be integrated to develop novel precision immuno-oncology platforms. In addition, development of functional assays to evaluate response and resistance to ICI therapy and novel combinations may require bio-engineering expertise to appropriately model the native tumour immune microenvironment (Hirt *et al*, 2014). In the short-term, composite biomarkers (e.g., CD8 T-cell abundance+tumoural/stromal PD-L1 staining) will likely supplant individual biomarkers (e.g., PD-L1 staining), whereas next-generation molecular and/or functional diagnostics are in development. Ultimately, such precision approaches would be anticipated to identify specific therapies, or therapeutic combinations, to optimise clinical activity and durability of clinical response for individual patients.
